# Relationships between Size Distribution, Morphological Characteristics, and Viscosity of Cellulose Nanofibril Dispersions

**DOI:** 10.3390/polym14183843

**Published:** 2022-09-14

**Authors:** Gregory Albornoz-Palma, Daniel Ching, Andrea Andrade, Sergio Henríquez-Gallegos, Regis Teixeira Mendonça, Miguel Pereira

**Affiliations:** 1 Departamento de Ingeniería Química, Universidad de Concepción, Concepción 4030000, Chile; 2 Facultad de Ciencias Forestales, Universidad de Concepción, Concepción 4030000, Chile; 3 Centro de Biotecnología, Universidad de Concepción, Concepción 4030000, Chile; 4 Unidad de Desarrollo Tecnológico (UDT), Universidad de Concepción, Coronel 4190000, Chile

**Keywords:** cellulose nanofibrils, viscosity, dilute dispersion, semi-dilute dispersion

## Abstract

Rheological parameters of cellulose nanofibril dispersions (CNF) are relevant and commonly used as quality control for producing of this type of material. These parameters are affected by morphological features and size distribution of the nanofibrils. Understanding the effect of size distribution is essential for analyzing the rheological properties, viscosity control, performance of CNFs, and potential dispersion applications. This study aims at comprehending how the morphological characteristics of the CNFs and their size distribution affect the rheological behavior of dispersions. The CNF dispersions were fractionated by size, obtaining six fractions of each, which were analyzed for their morphology and rheology (viscosity, intrinsic viscosity). In the dilute region, the viscosity and intrinsic viscosity behavior of CNF dispersions are linear concerning the size distribution present in the dispersion. In the semi-dilute region, the size of the fibrils and the fiber aggregates have a relevant effect on the viscosity behavior of CNF dispersions, which are satisfactorily related (R^2^ = 0.997) using the rule of logarithmic additivity of the dispersion viscosities of size fractions.

## 1. Introduction

Cellulose nanofibrils (CNFs) are semiflexible nano-objects [1,2]. They are produced as highly viscous aqueous dispersions at a low solids content [3]. These viscous dispersions are formed due to the hygroscopic nature of cellulose, the high aspect ratio, and the high specific surface area of nanofibrils. Together, they result in strong interconnections at low concentrations [4]. The average length of CNFs is estimated to be several micrometers, and the estimated aspect ratio of these nano-objects is in the order of hundreds [5]. This gives it a somewhat percolation threshold; accordingly, they have an efficient ability to form a rigid network [6].

The mechanical fibrillation process produces a non-homogeneous nanomaterial that contains fibers, macrofibrils, and nanofibrils [7]. Fibrils are usually characterized using imaging methods such as field emission scanning electron microscopy (FE-SEM), Atomic force microscopy (AFM), or Transmission electron microscopy (TEM), since they are an essential part of the morphological characterization of CNFs [8,9]. However, one of the problems when using these techniques is the heterogeneity of the nanomaterial and their reduced field of view, which introduces a subjective pre-selection of small areas containing nano-objects [7], thus possibly leading to errors in its characterization. Therefore, for the CNF characterization, it is necessary to conduct a series of complementary analyzes (fibrillar yield, specific surface, laser diffraction, among many others) that allow their correct characterization.

The material’s heterogeneity and size distribution affect the final rheological properties of the suspensions. Therefore, CNF dispersion flow measurements in the dilute region have been used to assess the morphological characteristics of CNFs. To this extent, the behavior and rheological characteristics of the dispersions can deliver relevant information about the morphology of CNFs. In the dilute region, intrinsic viscosity is one of the relevant transport properties [10]. In their studies, Tanaka et al. [11] and Albornoz-Palma et al. [12] established that the intrinsic viscosity of mechanical, enzymatic, and chemical CNF dispersions could be specifically related to the aspect ratio of nanofibrils, regardless of the flexibility of CNFs.

Additionally, the viscosity of CNF dispersions depends strongly on their concentration. Furthermore, it is considered one of the most critical transport properties. Generally, there are two different regions regarding concentration the dilute region, where interaction effects are not significant (Newtonian behavior) [1]: the semi-dilute region and the concentrated region. In the semi-dilute and concentrated regions, the rheological properties are often dominated by the interactions or aggregation and entanglements, usually affected by the surface charges of the fibrils, the functional groups, and their morphology [13].

In addition, the viscosity of CNF dispersions is much more difficult to predict than the viscosity of mixtures with polymeric components. Above the critical concentration [14], CNF dispersions have the following characteristics: (1) viscoelastic behavior [15,16]; (2) shear thinning, that is, their viscosity decreases with an increase in the shear rate [1]; (3) yield stress [17], and (4) thixotropic behavior [18], namely, its viscosity decreases over time when applied a constant shear rate [19]. All of these are caused by agglomeration and flocculation within the fibrillar matrix and the changes it presents [20]. A possible simple way to calculate the viscosity of CNFs is using the rule of logarithmic additivity [21] of the dispersion viscosities of size fractions.

The viscosity of CNF dispersions is essential to understanding and providing an idea of the morphological properties of CNFs since it reflects their flow behavior. Moreover, it shows its potential uses at an industrial level [22,23,24,25], such as rheology modifiers, composites, applications coating, polymer reinforcement, and 3D printing of hydrogels, among several others [26,27,28]. In addition, viscosity is a measure of the quality of the nanomaterial [29], which depends on the size distribution, the morphological characteristics, and the interactions between fibrils of different sizes. Therefore, these parameters are crucial to predicting the viscosity of the nanomaterial. For this reason, this study aims to explore relationships between the morphological properties, size distribution, and viscosities of CNF in dilute and semi-dilute dispersions.

## 2. Materials and Methods

### 2.1. Materials

CNF dispersions were produced from bleached hardwood Kraft pulp (90% Eucalyptus globulus and 10% Eucalyptus nitens) supplied by CMPC Pulp S.A. (Santa Fe Mill). The chemical composition of the pulp was 79 ± 1% cellulose, 20 ± 1% hemicellulose, and 0.5 ± 0.1% lignin. The physical characteristics of the fibers were 0.74 ± 0.04 mm in length and 18.5 ± 0.5 μm in diameter (Fiber tester L&W).

### 2.2. Preparation of CNF Dispersions

The pulp was enzymatically pretreated using the 1.2% Maximyze 2566 enzyme. The pretreatment was carried out at 10% (*w*/*w*) pulp consistency for 60 min at 35 °C, and with constant agitation. Subsequently, the enzyme was denatured at 80 °C for 20 min. Then, the pulp underwent a mechanical pretreatment using a PFI mill with 50,000 revolutions, followed by fiber disintegration through different numbers of passes in a GEA Niro Soavi homogenizer (Panda Plus 2000) at pressures between 600 and 800 bar and consistency of 0.5% (*w*/*w*) in distilled water as solvent. As a result, seven CNF dispersions with 0, 1, 2, 4, 7, 10, and 15 passes through the homogenizer were produced at a consistency of 0.5% (*w*/*w*), and approximately pH 6 was produced.

### 2.3. Size Distribution of CNF Dispersions

Samples of each CNF dispersion were prepared at a consistency of 1 (g/L). Each sample went through different meshes using the Williams Precision Freeness Tester filtration system with 45 (707 μm), 60 (300 μm), 100 (149 μm), 200 (74 μm) and 400 (37 μm) mesh. For each sample, a consecutive filtration sequence was performed. The mass retained in each mesh was diluted and filtered again to achieve good separation. For each sample, the dry mass retained in each mesh was quantified using the gravimetric method, and the CNF dispersions corresponding to each fraction by size were obtained.

### 2.4. Morphological Characteristics

The average length of CNF dispersions was measured from dispersions at 0.04% (*w*/*v*) consistency using an S3500 Laser Diffraction Particle Size Analyzer (Microtrac Inc., York, PA, USA) (refractive index: 1.54 [30]). Each sample was sonicated for 60 s in the equipment before measuring to eliminate entanglements and aggregates that could have formed. The particle size measurement range of the S3500 was from 0.02 to 2800 microns. Its configuration corresponds to a Tri-Laser system (three red laser diodes) that provides a reliable and repeatable particle size analysis using the principle of Modified Mie calculations for non-spherical particles [31,32]. Four different samples were measured for each type of CNF, with five repetitions each. With this characterization method, the length of the CNFs was defined as the spherical equivalent diameters that occupy the same volume as the irregular fibrils.

The average width was measured from CNF dispersions at 0.1% (*w*/*w*) consistency, measuring about 220–240 fibrils by simple random sampling using micrographs of transmission electron microscopy (TEM) at different scales (magnification: ×2.0 k, ×5.0 k, ×8.0 k, ×15.0 k, and ×25.0 k) obtained by using the Hitachi TEM system with an accelerating voltage of 80.0 kV. The dispersion was placed on a copper grid and dried for 10 min at room temperature. For each type of CNF, more than 20 micrographs were used, and four different samplings were conducted. TEM micrographs were processed through the image analysis software ImageJ.

The aspect ratio of the CNFs was calculated by dividing the average length of the CNFs measured by Laser Diffraction and the average width of the CNFs measured by simple random sampling using micrographs of TEM.

The radius of gyration was calculated using the wormlike chain model [10] using the experimental average lengths and widths and a flexibility parameter (ϵ) equal to 0.32 [12].

### 2.5. Shear Viscosity Measurement

The viscosities of CNF dispersions were measured using a Brookfield LVDV-I + viscometer with a ULA (Ultra Low Adapter) spindle. The configuration was set to a double-cylinder geometry (cup radius: 1.37 cm; spindle radius: 1.25 cm). Dispersions for each sample were prepared at different concentrations (0.02% (*w*/*v*) to 0.3% (*w*/*v*)). Viscosity values obtained from the CNF dispersions with a concentration under the critical concentration were used to determine the intrinsic viscosity. Each of the samples was heated in a Julabo SW22 thermal bath at 23 °C for two h before the measurement. Subsequently, all samples were shaken for 2 min in an MX-S Mlab Scientific vortex before measuring. Samples whose concentrations were lower than the critical concentration (dilute region) did not show thixotropic behavior and reached a steady state in less than 20 s. The samples with the highest concentration (semi-dilute region) presented thixotropic behavior, reaching a steady state in less than 4 min. From the beginning of the shear, the measurement time for the viscosity of CNF dispersions was 4 min. The measurement conditions were 23 °C with a shear rate of 73.38 s^−1^.

### 2.6. Critical Concentration

The critical concentration was obtained from the changes of specific viscosity as a function of the CNF concentrations, according to Morris et al. [33].

### 2.7. Data Analysis

The statistical analysis of the data was carried out using the Statgraphics Centurion XVII program.

## 3. Results and Discussion

### 3.1. Morphological Characteristics of CNFs

Table 1 shows the morphological characteristics and intrinsic viscosity of the CNF dispersions with a different number of passes through the homogenizer [12]. As the applied mechanical treatment duration increases, the average length and width of the CNFs tend to decrease. This is caused by the deconstruction of the cell walls in plant cells, where, at a higher level of fibrillation, nano-objects are produced shorter (transversal deconstruction) and thinner (longitudinal deconstruction). In addition, the distributions tend to be more homogeneous (decrease in the standard deviation of the samples) as a result of the higher level of fiber and macrofibril deconstruction at a higher duration in the mechanical treatment.

The radius of gyration corresponds to a parameter characterizing the particle size of any shape [34]. Table 1 shows that as the number of passes through the homogenizer increases, the size of the nano-objects present in the dispersion decreases. Hence, the radius of gyration decreases. In contrast, the aspect ratio increases with the rise in the duration of the mechanical treatment because the longitudinal deconstruction of the fibers is more severe than the transversal deconstruction, which produces increasingly thin nano-objects with minor variations in length. Furthermore, the previously mentioned does not occur between the levels of 7 and 10 passes, where the average widths of the fibrils are statistically equal. In contrast, the length of the fibrils changes significantly.

Additionally, the intrinsic viscosity can be thought of as the hydrodynamic volume occupied by a unit mass of the particles present in dilute dispersion [35,36,37], where the hydrodynamic volume corresponds to the volume of a hydrodynamically equivalent sphere [34]. Since the hydrodynamic volume is proportional to the radius of gyration [35,38,39,40], it is expected that as the duration of treatment increases, the hydrodynamic volume, and the radius of gyration of individual fibrils decrease. However, it is observed in Table 1 that the intrinsic viscosity increases, indicating that the set of larger fibrils has a lower hydrodynamic volume per unit mass than the set of smaller fibrils. The above conveys the relevance of the morphology of the fibrils in the rheological behavior of CNF dispersions in the dilute region (without interaction). Therefore, this should be considered in the flow analyses in semi-dilute and concentrated regions to study real interaction effects.

### 3.2. Size Distribution

To study the effects generated by the size distribution in the rheological properties of each CNF dispersion in the dilute and semi-dilute regions, the mass fractions of the different size fractions (by volume) present in the dispersions were determined.

**Figure 1:**
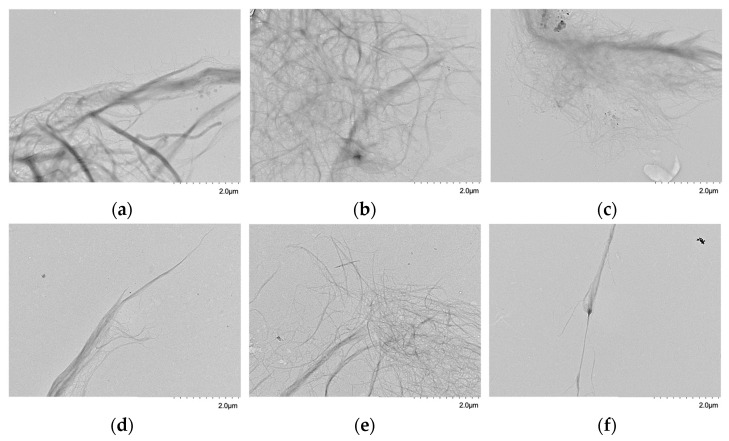
TEM micrographs of CNFs fractioned by size: (**a**) 45 mesh, (**b**) 60 mesh, (**c**) 100 mesh, (**d**) 200 mesh, (**e**) 400 mesh, and (**f**) under 400 mesh (scale: 2 μm, magnification: ×5.0 k).

Table 2 shows the size distributions of the different CNF dispersions. It can be observed that as the level of fibrillation increases, the mass percentage of smaller fibrils also increases significantly. Before the mechanical homogenization process, the percentage of large structures is significant, which mainly includes tangles between residual fibrils and nanofibrils (Figure 1a), and therefore have large volumes that are retained in the early stages of fractionation. However, with 0 passes, there is a high percentage of nanoobjects, corresponding to 26.4% (less than 100 mesh) (Table 2 and Table 3), where 13% corresponds to structures with widths smaller than 50 nm (less than 400 mesh). This result is consistent with the distributions shown in Albornoz-Palma et al. [12] for the dispersions. As the number of passes through the homogenizer increases, two effects are observed (Table 2). The first is associated with the deconstruction of residual fibers (widths higher than 100 nm), which decrease significantly in size, from 73.6% in 0 passes to 6.6% in 1 pass and implies that the mechanical process severely affects these structures. The second one relates to the increase in the homogeneity of the samples. This is caused by the deconstruction of the residual fibrils and macrofibrils, whose minimum possible width depends on the size of the homogenizer chamber [22].

The transmission electron microscopy (TEM) micrographs (Figure 1) of the different size fractions allowed us to observe the change in the structures produced during the homogenization process. With a 45 mesh, the retained structures correspond to large residual fibers strongly entangled with nanofibrils or partially fibrillated, which causes the entanglement to have a large volume. With a more refined mesh (60, 100, and 200), the structures have a smaller size, and the residual fibrils or macrofibrils have a higher level of fibrillation and a lower level of entanglement. This allows for a higher level of separation and, therefore, a lower volume in dispersion. The fibrils in the narrowest separations (400 mesh and low 400 mesh) are highly fibrillated, meaning their fibrils are effectively separated or with a lower volume of entanglement.

The morphology of the fibrils present in the different size fractions is shown in Table 3 and Figure S1 (Supplementary Material). As expected, the average lengths and widths tend to decrease as finer meshes are used. The standard deviations of the length of CNFs of all the samples are small (coefficient of variation less than 60%) compared to those of the original samples (coefficient of variation greater than 70%) (Table 1). This implies that the separation process achieved a relatively homogeneous distribution in sizes in the stages of fractionation.

Regarding the intrinsic viscosity and aspect ratio of the fractionated samples (Table 3), these increase as the mesh becomes finer. The previously mentioned implies that the hydrodynamic volume per unit mass occupied by a large entanglement is less when the system is fibrillated. In addition, as the level of fibrillation increases, the parameter that changes the most is the width, causing the aspect ratio to increase. Between the levels of 7 and 15 passes under a 400 mesh, the shortening of the fibrils was more significant than their transversal deconstruction (decrease in aspect ratio), which generated a decrease in the effective volume occupied by the fibrils per unit mass, producing a lower intrinsic viscosity. This behavior was previously evidenced for the samples corresponding to 7, 10, and 15 passes through the homogenizer, whose fractionation of the samples with 7 and 15 passes corroborated the trends mentioned above (Table 1). The determination of the intrinsic viscosity of the different CNF fractions is shown in Figure S2 (Supplementary Material).

The critical concentration (see Table 3 and Figure S3 in the Supplementary Material) is strongly influenced by the morphology resulting from the mechanical homogenization process, mainly associated with the hydrodynamic volume occupied by the nano-objects present in the dispersions. Highly fibrillated systems occupy a larger volume in the dispersion at a fixed concentration level. As the concentration increases, the probability of contact between the fibrils increases. Therefore, the formation of aggregates or flocs is favored, which results in the decrease of the dilute region and, consequently, the decrease in the critical concentration. As observed between levels of 7 and 15 passes under 400 mesh, the volume occupied by the fibrils is lower, and the probability of contact decreases, generating an increase in critical concentration.

Flocculation and entanglement are different in terms of dispersion. Flocculation corresponds to the instability of dispersion induced by particle–particle collisions under shear, that is, hydrodynamic motions (orthokinetic aggregation) [41,42]. Although shear-induced flocculation may, at the same time, result in induced entanglement, entanglement can also be caused simply by mechanical interference [43].

In a previous work [12], correlations were established between the intrinsic viscosity ([η]) and the aspect ratio (p) of the CNFs (ρ[η] = 0.051p^1.85^, where ρ is the density of CNFs), and between the critical concentration (c*) and the aspect ratio of the CNFs (c* = 67(1⁄p^2^)). If we compare the experimental results with the predicted ones, a maximum variation of 5.8% for average width and 14.8% for the critical concentration was obtained. These results allow corroborating the correlations.

### 3.3. Dilute and Semi-Dilute Regions

To verify the values of the mass fractions obtained for each CNF dispersion (Table 2), the value of the average width of each sample was determined as a linear weighting between the mass fraction and the average width corresponding to each fraction and compared with the experimental average width (Equation (1)):

(1) $${\bar{d}}_{pred}=\underset{i}{\sum }x_{i}{\bar{d}}_{i}=1.04\;{\bar{d}}_{exp}$$

where ${\bar{d}}_{pred}$ is the predicted average width (nm), $x_{i}$ is the mass fraction of the *i*-th fraction, ${\bar{d}}_{i}\;$ is the average width of the *i*-th fraction (nm), and ${\bar{d}}_{exp}$ is the experimental average width (nm).

**Figure 2:**
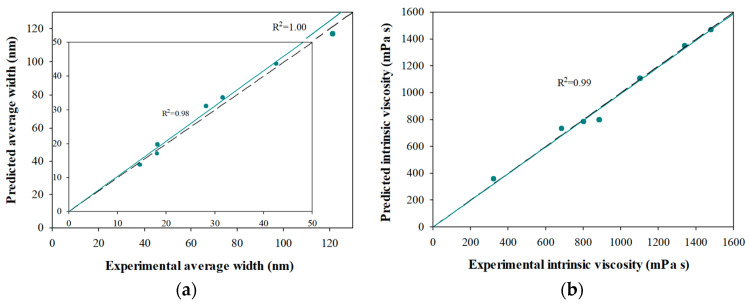
(**a**) Pareto chart for average width and (**b**) intrinsic viscosity by fractions in size.

The linear regression between the variables presents a slope corresponding to the unit (Figure 2a), whose coefficient of determination is 98%, when not considering 0 passes, and 100% when all the dispersions are included. This verifies the values of the mass fractions obtained from the different dispersions.

In systems with a wide size distribution, intrinsic viscosity can be determined as the linear contribution between the mass fraction and intrinsic viscosity of each fraction [44]:

(2) $${\left[\eta \right]}_{pred}=\underset{i}{\sum }x_{i}{\left[\eta \right]}_{i}=0.99\;{\left[\eta \right]}_{exp}$$

where is ${\left[\eta \right]}_{pred}\;$ the predicted intrinsic viscosity (mL/g), $x_{i}\;$ is the mass fraction of the i-th fraction, ${\left[\eta \right]}_{i}$ is the intrinsic viscosity of the *i*-th fraction (mL/g), and ${\left[\eta \right]}_{exp}$ is the experimental intrinsic viscosity (mL/g).

The linear regression between the variables (Equation (2)) presented a unitary slope, with a coefficient of determination of 99% (Figure 2b). This result proves that the intrinsic viscosity of CNF dispersions measured in the dilute region is not affected by aggregation effects. Therefore, since there is a direct relationship between the intrinsic viscosity of the CNF dispersions and the aspect ratio of the CNFs, it is possible to predict and calculate this rheological parameter from the morphological characterization of its constituents or vice-versa.

Additionally, when establishing the direct relationship between morphology, size distribution, and rheological properties of CNF dispersions in the dilute region, this information should be considered when analyzing the behavior of dispersions in the semi-diluted region.

**Figure 3:**
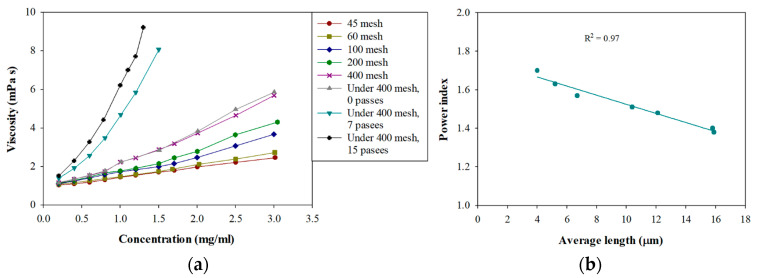
(**a**) Variation in the infinite shear viscosity according to the concentration of the different fractions of CNFs. (**b**) Variation in power index according to the average length of the CNFs.

The dependence on viscosity and concentration is relevant in the different concentration regions. The usual form to report viscosity is by specific viscosity. In the dilute region, the dependence between these variables is linear. In contrast, in the semi-dilute region, the dependence of the specific viscosity ($\eta_{sp}$) follows a power-law model with the concentration (c): $\eta_{sp}\sim c^{n}$ [14]. By adjusting the specific viscosity of the different fractions with the concentration (above the critical concentration), the power index values (n) for each fraction were obtained (Table 3). When the fraction is finer, the value of the power index increases. This is because the higher the level of fibrillation, the levels of interaction between the fibrils increase and the formation of fibril flocs is favored. Flocs are aggregates that are usually very open, which produce a greater resistance to flow and, therefore, a more significant increase in viscosity [45,46] (Figure 3a).

When the power index was related to the morphological properties of the CNFs, a strong dependence on the average length was observed. Figure 3b shows the relationship obtained between the average length of the CNFs and the power index. This correlation relates to a linear relationship whose coefficient of determination is 97%, indicating that the correlation obtained is adequate. This simple relationship between the power index (rheological parameter) and the average length of the CNFs (morphological parameter) allows for predicting the formation of fibrillar networks with increasing concentration, permitting comparison of CNF dispersions and their potential uses. Furthermore, Silveira et al. [47] showed a similar relationship between fibers from eucalyptus and pine-bleached Kraft pulp.

Due to the complexity of describing the viscosity of CNF dispersions, the dependence of the shear rate is generally separated from other variables such as temperature, concentration, solvent, and entanglement, among others [21,48]. Therefore, a nonlinear regression analysis was performed with two interactions using Statgraphics Centurion XVII program to study the contribution of the other variables to the viscosity of each sample (CNF and fraction). The variables considered were concentration, average length, average width, and aspect ratio (details are presented in Table S1 in the Supplementary Material).

The Analysis of Variance (ANOVA) showed that three parameters are significant in explaining the behavior of the viscosity of the CNF dispersions (*p*-value < 0.05). The parameters are: (1) the concentration, (2) the average length, and (3) the aspect ratio. In addition, the regression obtained showed the presence of two interactions (Figure S4, Supplementary Material), a positive one, between the concentration and the aspect ratio of the CNFs, and a negative one, between the average length and aspect ratio of the CNFs. Both interactions are within the viscosity ranges corresponding to the semi-dilute region; therefore, combined effects must be considered.

**Figure 4:**
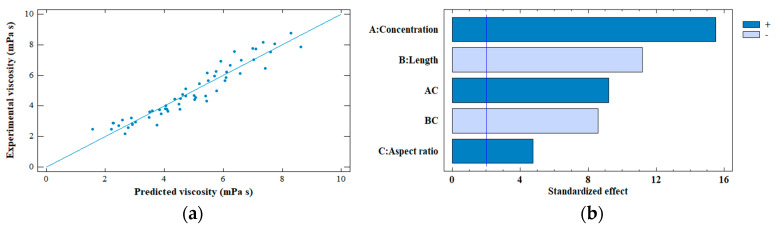
(**a**) Predicted viscosity versus experimental viscosity, and (**b**) Standardized Pareto chart for the viscosity of CNF dispersions. AC: Concentration-Length interaction, and BC: Length-Aspect ratio interaction.

As for the statistical parameters, the regression has an adjusted coefficient of determination of 91% and a Durbin-Watson statistic of 1.7 (*p* = 0.06). The above indicates that the fitted model is good and that there is no serial autocorrelation between the residues. The experimental and predicted viscosity values are shown in Figure 4a.

The relevant variables that explain the viscosity of CNF dispersions in the semi-dilute region are effectively related to the viscosity dependence variables of polymer solutions (solvent, concentration, entanglement, and molecular weight) [48], where the aspect ratio replaces the molecular weight, as shown in Tanaka et al. [11] and Albornoz-Palma et al. [12], and the entanglements are substituted by the length of the CNFs, since entanglements between particles occur in semi-flexible and elongated materials [43]. As shown in Figure 3b, the average length of the CNFs is related to the formation of fibrillar networks (power index).

Figure 4b shows the Pareto diagram, where the concentration and the average length of the CNFs are the parameters that have the highest effect on the viscosity of the dispersions, and the aspect ratio has the least effect. Concentration and aspect ratio have positive effects on viscosity, whereas the length of CNFs has a negative effect, meaning that the greater the length, the viscosity tends to decrease.

The viscosity at low concentrations (dilute region) is dominated by the resistance generated by the fibrils and the entanglements present in the dispersion. Therefore, the interaction effects are neglected. At a higher concentration (semi-dilute region), the resistance is mainly granted by the flocs formed in the dispersion, which depend on the nature of the dispersion, the solvent, the concentration, the shear rate, and the size distribution, among others.

To model the viscosity of CNFs, the rule of logarithmic additivity [21,49] of the dispersion viscosities of size fractions was used:

(3) $$\log\eta =\underset{i}{\sum }x_{i}\log\eta_{i}$$

where η is the viscosity of the dispersion (mPa s), $x_{i}\;$ is the mass fraction of the *i*-th fraction, and $\eta_{i}$ is the viscosity of the *i*-th fraction (mPa s).

**Figure 5:**
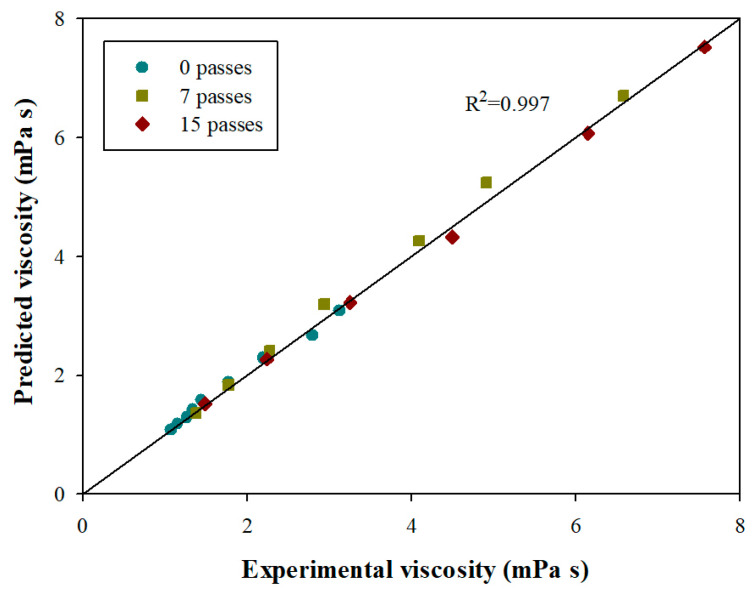
Predicted viscosity versus experimental viscosity of CNF dispersions in the dilute and semi-dilute regions.

The viscosity results predicted (by the logarithmic mixing rule) as a function of experimental viscosities are presented in Figure 5.

In the dilute region, where the interactions are not significant, the viscosity of the CNFs could be modeled simply as the contribution between the mass fraction and the viscosity of each size fraction, using a logarithmic mixture rule (or even using a mixture linear ideal [50]). In this region, the probability of collisions between the fibrils is scarce or almost zero, so the viscosities in this concentration range do not differ significantly (Figure 3a) because the resistance to flow depends mainly on the shape of the fibrils.

In the semi-dilute region, the interactions between fibrils are significant. Furthermore, the aggregates formed depend on the size distribution of fibrils, generating flocs in the CNF dispersions different from those quantified in the size fractions. Despite the above, the viscosities of the CNFs could be modeled using the logarithmic mixing rule between the viscosity and mass fraction of the different size fractions (Figure 5), but not with an ideal linear mixing.

The viscosity values predicted by the linear weighting between the viscosity and mass fraction of the different size fractions are more significant than the experimental values. Moreover, they are caused by the size and probability of contact or collisions between the fibrils. When the samples are highly fibrillated, the volume occupied per unit mass by nanofibrils or fibril aggregates is higher, due to the high probability of contact between the fibrils, which leads to higher levels of interaction. This favors flocculation and, therefore, produces a higher resistance to flow. When there is more than one size present (size ranges), flocculation no longer occurs only with fibrils or fibril aggregates of similar hydrodynamic volume, but also with fibrils or fibril aggregates of lower hydrodynamic volume per unit mass, which results in lower flow resistance and, therefore, a lower viscosity. This new floc configuration was satisfactorily described by logarithmic weighting, where this weighting allows a smaller mean to a more heterogeneous distribution.

Finally, the coefficient of determination of the viscosity model in the dilute and semi-dilute region was 99.7%, which indicates that the model is satisfactory.

## 4. Conclusions

In the dilute region, it was shown that the rheological behavior of the viscosity and intrinsic viscosity of CNF dispersions is satisfactorily explained by linear contribution models between their different size fractions, which shows, indeed, that the parameters were not affected by the interactions. In the semi-dilute region, the size of the fibrils and the fiber aggregates have a relevant effect on the viscosity behavior of CNF dispersions. In addition, it was shown that the parameters that mainly affect the viscosity are the concentration of the dispersion, the aspect ratio, and the average length of CNFs, with different proportions, concentration, and average length being the most important. The viscosity of the CNF dispersions was adequately modeled by the rule of logarithmic additivity of the viscosities of size fraction (R^2^ = 0.997), where the smaller weighting of means for more heterogeneous distribution reflected the flocculation of the fractions within the dispersion of CNF.

## Figures and Tables

**Table 1:** Morphological characteristics and intrinsic viscosity of CNF dispersions (adapted from Albornoz-Palma et al. [12]).

No.	Average Width ^(a)^ (nm)	S.D. of the Sample ^(a,b)^ (nm) (Coefficient of Variation ^(a)^ (%))	Average Length ^(a)^ (μm)	S.D. of the Sample ^(a)^ (μm) (Coefficient of Variation ^(a)^ (%))	Radius of Gyration (μm)	Aspect Ratio ^(a)^	Intrinsic Viscosity ^(a)^ (mL/g)
0P	121.4 ± 1.0	68.1 (56)	17.7 ± 0.4	15.9 (90)	3.87	145.5	323.2
1P	42.6 ± 0.4	22.3 (51)	8.9 ± 0.4	6.3 (71)	2.01	210.1	684.4
2P	31.6 ± 0.2	13.0 (41)	7.7 ± 0.4	5.3 (70)	1.73	243.8	801.6
4P	28.2 ± 0.2	10.6 (38)	7.5 ± 0.2	5.3 (70)	1.68	264.8	885.2
7P	18.2 ± 0.2	6.3 (35)	5.9 ± 0.3	3.7 (63)	1.32	325.4	1481.6
10P	18.1 ± 0.3	6.0 (34)	5.0 ± 0.4	3.5 (69)	1.13	278.7	1102.5
15P	14.6 ± 0.1	4.0 (28)	4.4 ± 0.8	3.3 (75)	0.99	304.6	1339.8

^(a)^ Albornoz-Palma et al. [12]; ^(b)^ Normal distribution by the central limit theorem.

**Table 2:** Mass fractions resulting from fractionation by the size of the different CNF dispersions.

Mesh (Width)	0 Passes	1 Pass	2 Passes	4 Passes	7 Passes	10 Passes	15 Passes
45 (180.1 nm)	36.6%	2.3%	-	-	-	-	-
60 (100.8 nm)	37.0%	4.3%	0.6%	-	-	-	-
100 (84.3 nm)	7.0%	6.1%	2.1%	0.7%	0.1%	-	-
200 (56.1 nm)	6.4%	8.7%	5.2%	2.1%	0.4%	0.4%	0.2%
400 (44.9 nm)	3.4%	18.0%	14.2%	11.3%	12.0%	11.3%	1.9%
Under 400	9.6%	60.6%	77.9%	85.9%	87.5%	88.3%	97.9%

**Table 3:** Morphological and rheological characteristics of the different fractions by size.

Mesh	Average Width (nm)	S.D. of the Sample ^(a)^\nm (Coefficient of Variation (%))	Average Length (μm)	S.D. of the Sample (μm) (Coefficient of Variation (%))	Aspect Ratio	Critical Concentration (mg/mL)	Intrinsic Viscosity (mL/g)	Power Index ^(c)^
45	180.1 ± 0.5	64.6 (36)	16.9 ± 0.4	10.2 (60)	93.6	7.6 ^(b)^	134.0	-
60	100.8 ± 0.5	47.7 (47)	15.9 ± 0.5	9.5 (60)	158.0	2.3	356.9	1.38
100	84.3 ± 0.4	30.8 (37)	15.8 ± 0.1	8.6 (54)	187.9	2.1	502.2	1.40
200	56.1 ± 0.3	26.2 (47)	12.1 ± 0.7	7.2 (59)	214.9	1.6	596.0	1.48
400	44.9 ± 0.3	19.6 (44)	10.4 ± 0.5	5.1 (49)	230.8	1.1	786.6	1.51
Under 400, 0 passes	28.3 ± 0.2	12.7 (45)	6.7 ± 0.1	3.4 (51)	237.2	1.0	807.7	1.57
Under 400, 7 passes	16.1 ± 0.3	7.2 (45)	5.2 ± 0.3	2.3 (44)	323.7	0.57	1567.9	1.63
Under 400, 15 passes	13.1 ± 0.2	3.4 (26)	4.0 ± 0.3	2.0 (50)	306.2	0.62	1361.5	1.70

^(a)^ Normal distribution by the central limit theorem; ^(b)^ Calculated by equation c* = 67(1⁄p^2^) (Albornoz-Palma et al. [12]); ^(c)^ Calculated by equation η_sp_ = ac^n^ (Krishnan, [14]).

## Data Availability

Not applicable.

## Supplementary Materials

The following supporting information can be downloaded at: https://www.mdpi.com/article/10.3390/polym14183843/s1, Figure S1: Width distributions of CNFs. *TEM images are available contacting the corresponding author.*; Figure S2. Variation in the reduced viscosity according to the concentration of CNFs in the dilute region; Figure S3. Variation in the specific viscosity, *η_sp_*, according to the concentration, c (g/mL), of the different fractions of CNFs. All the coefficients of determination of the regressions were higher than 0.98; Figure S4. Interaction plot for the viscosity of CNF dispersions. *A: Concentration, B: Length, and C: Aspect ratio*; Table S1: Regression coefficient for viscosity.
