# Supplementary material for: Relationships between Size Distribution, Morphological Characteristics, and Viscosity of Cellulose Nanofibril Dispersions
Source: Polymers (Basel). 2022 Sep 14;14(18):3843. doi: 10.3390/polym14183843 (PMC9506213; doi:10.3390/polym14183843)
Supplement: Supplementary file 1 [file polymers-14-03843-s001.zip › polymers-1910835-supplementary.pdf]

Supplementary material for

Article

Relationships between Size Distribution, Morphological Characteristics, and Viscosity of Cellulose Nanofibril Dispersions

Gregory Albornoz-Palma [1,*], Daniel Ching [1], Andrea Andrade [2], Sergio Henríquez-Gallegos [2], Regis Teixeira Mendonça [2,3] and Miguel Pereira [1,4]

[1] Departamento de Ingeniería Química, Universidad de Concepción, Concepción 4030000, Chile
[2] Facultad de Ciencias Forestales, Universidad de Concepción, Concepción 4030000 Chile
[3] Centro de Biotecnología, Universidad de Concepción, Concepción 4030000, Chile
[4] Unidad de Desarrollo Tecnológico (UDT), Universidad de Concepción, Coronel 4190000, Chile
* Correspondence: gralbornoz@udec.cl

This file includes: Supplementary **Figures S1, S2, S3,** and **S4,** and **Table S1**

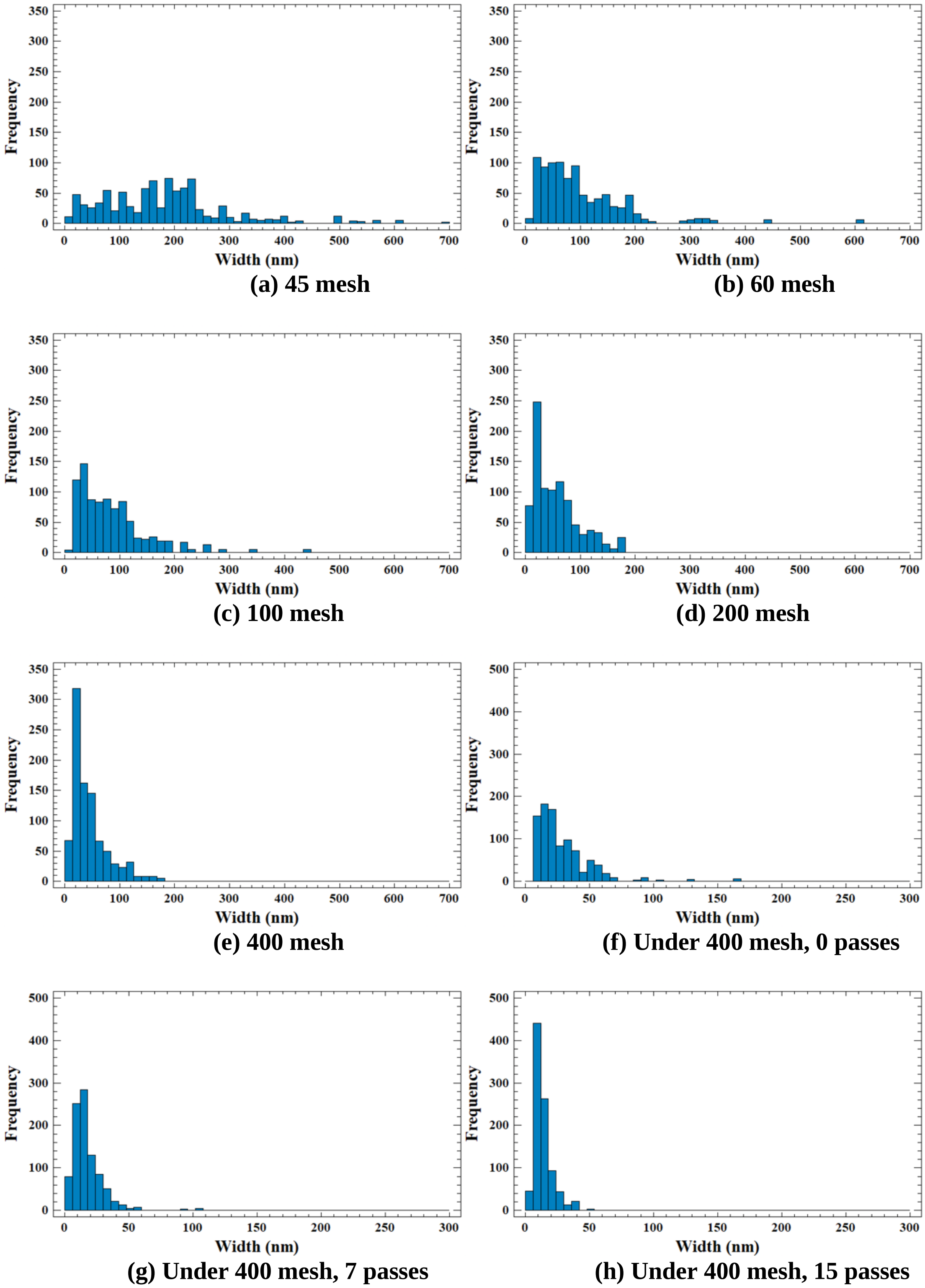

Figure S1. Width distributions of CNFs. *TEM images are available contacting the corresponding author*.

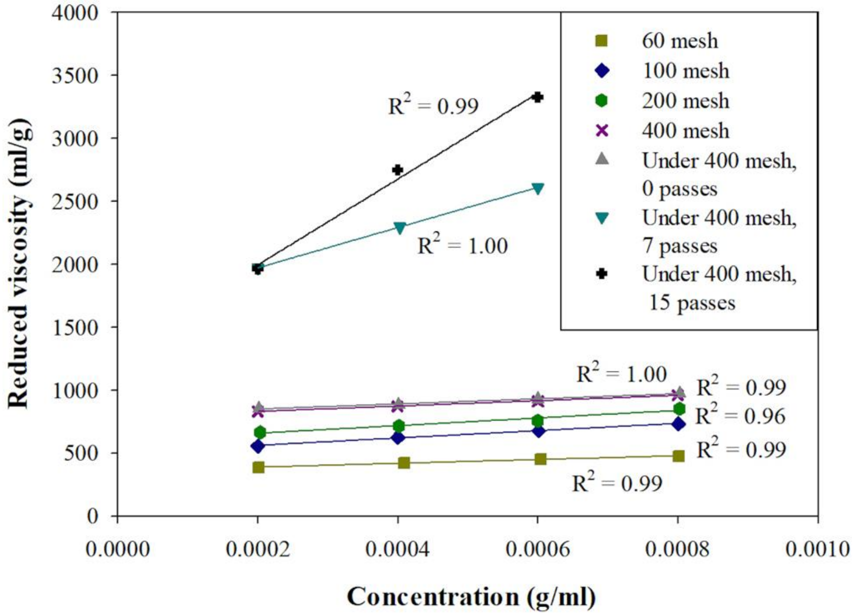

Figure S2. Variation in the reduced viscosity according to the concentration of CNFs in the dilute region.

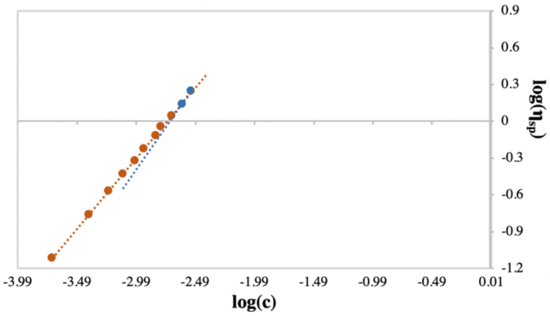

(a) 60 mesh

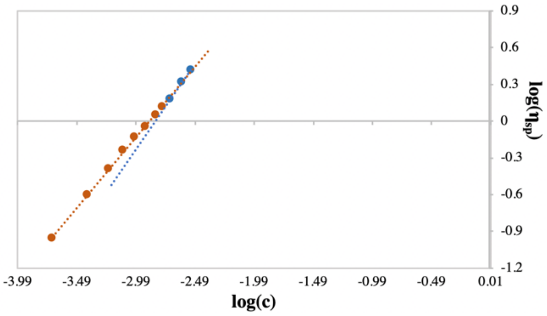

(b) 100 mesh

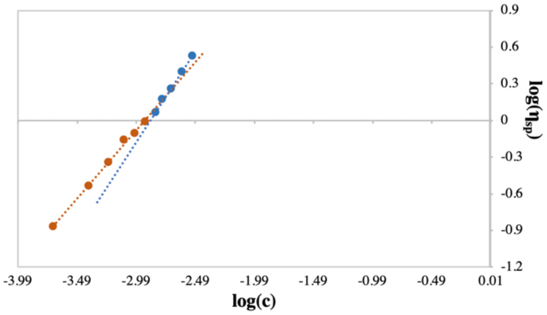

(c) 200 mesh

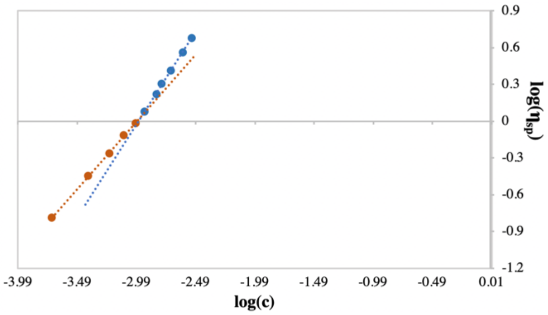

(d) 400 mesh

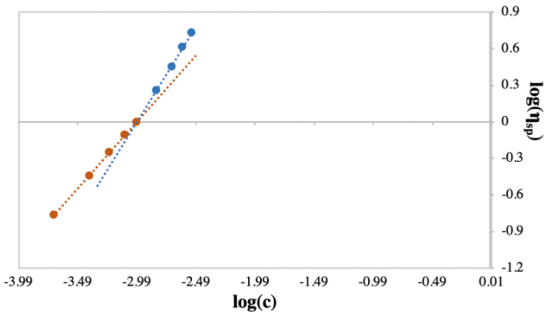

(e) Under 400 mesh, 0 passes

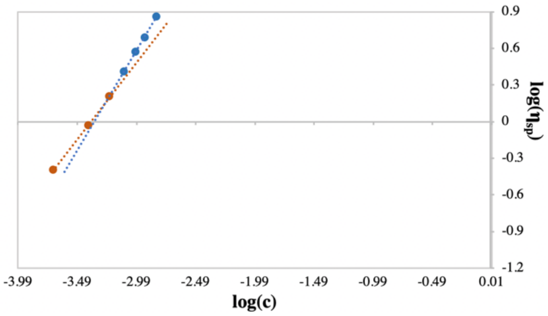

(f) Under 400 mesh, 7 passes

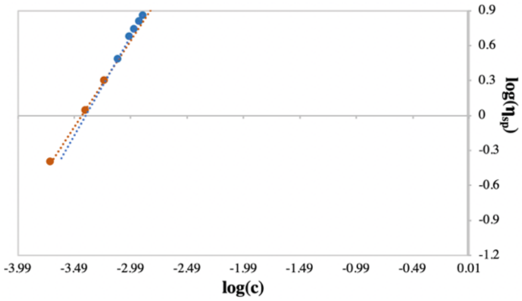

(g) Under 400 mesh, 15 passes

Figure S3. Variation in the specific viscosity, η_{sp}, according to the concentration, c (g/ml), of the different fractions of CNFs. All the coefficients of determination of the regressions were higher than 0.98.

Table S1. Regression coefficient for viscosity.

$$\eta = \eta_0 + a_1 \cdot c + a_2 \cdot L + a_3 \cdot p + a_4 \cdot c \cdot p + a_5 \cdot L \cdot p$$

Constant	Estimated coefficient
a_1	-20.88
a_2	0.6225
a_3	0.01453
a_4	0.2209
a_5	-0.005438

where η is the viscosity (mPa s), η_0 is the viscosity of the solvent (mPa s), c is the concentration (% g/ml), L is the average length (μm), p is the aspect ratio, and a_i is a constant.

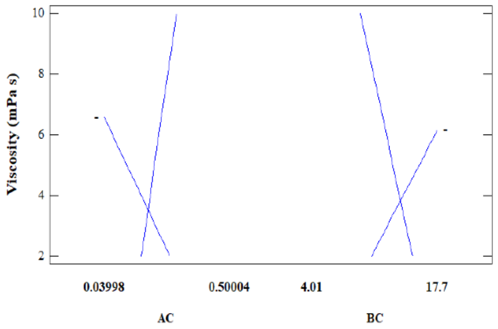

Figure S4. Interaction plot for the viscosity of CNF dispersions. *A: Concentration, B: Length, and C: Aspect ratio*
